# Non-invasive diagnosis of non-alcoholic fatty liver disease: Current status and future perspective^☆^

**DOI:** 10.1016/j.heliyon.2024.e27325

**Published:** 2024-02-29

**Authors:** Jia-Lan Wang, Su-Wen Jiang, Ai-Rong Hu, Ai-Wu Zhou, Ting Hu, Hong-Shan Li, Ying Fan, Ken Lin

**Affiliations:** a Graduate School of Wenzhou Medical University, Ningbo No. 2 Hospital, Ningbo, 315020, Zhejiang Province, China; b Precision Diagnosis and Treatment Center of Liver Diseases, Ningbo No. 2 Hospital, Ningbo, 315020, Zhejiang Province, China; c School of Medicine, Shaoxing University, Shaoxing, 31200, Zhejiang Province, China; d School of Medicine, Ningbo University, Ningbo, 315211, Zhejiang Province, China

**Keywords:** Non-alcoholic fatty liver disease, Non-alcoholic steatohepatitis, Biomarker, Non-invasive diagnosis

## Abstract

Non-alcoholic fatty liver disease (NAFLD) is the most common cause of chronic liver disease throughout the world. Hepatocellular carcinoma (HCC) and liver cirrhosis can result from nonalcoholic steatohepatitis (NASH), the severe stage of NAFLD progression. By some estimates, NAFLD affects almost one-third of the world's population, which is completely new and serious public health issue. Unfortunately, NAFLD is diagnosed by exclusion, and the gold standard for identifying NAFLD/NASH and reliably measuring liver fibrosis remains liver biopsy, which is an invasive, costly, time-consuming procedure and involves variable inter-observer diagnosis. With the progress of omics and imaging techniques, numerous non-invasive serological assays have been generated and developed. On the basis of these developments, non-invasive biomarkers and imaging techniques have been combined to increase diagnostic accuracy. This review provides information for the diagnosis and assessment of NAFLD/NASH in clinical practice going forward and may assist the clinician in making an early and accurate diagnosis and in proposing a cost-effective patient surveillance. We discuss newly identified and validated non-invasive diagnostic methods from biopsy-confirmed NAFLD patient studies and their implementation in clinical practice, encompassing NAFLD/NASH diagnosis and differentiation, fibrosis assessment, and disease progression monitoring. A series of tests, including 20-carboxy arachidonic acid (20-COOH AA) and 13,14-dihydro-15-keto prostaglandin D2 (dhk PGD2), were found to be potentially the most accurate non-invasive tests for diagnosing NAFLD. Additionally, the Three-dimensional magnetic resonance imaging (3D-MRE), combination of the FM-fibro index and Liver stiffness measurement (FM-fibro LSM index) and the machine learning algorithm (MLA) tests are more accurate than other tests in assessing liver fibrosis. However, it is essential to use bigger cohort studies to corroborate a number of non-invasive diagnostic tests with extremely elevated diagnostic values.

## Abbreviations

NAFLD: Non-alcoholic fatty liver disease
MAFLD: Metabolic dysfunction-associated steatotic liver disease
NAFL: Non-alcoholic fatty liver
NASH: Non-alcoholic steatohepatitis
HCC: Hepatocellular carcinoma
BMI: Body mass index
AST: Aspartate aminotransferase
ALT: Alanine aminotransferase
SCR: Serum creatinine
e-GFR: Estimated glomerular filtration rate
UA: Uric acid
LDL-TG: Low-density lipoprotein triglyceride
LDL-C: Low-density lipoprotein cholesterol
APAI1: Activated plasminogen activator inhibitor 1
IL -8: Anterleukins-8
CK18–F: Cytokeratin 18 fragment
PC-III: Serum procollagen type III
MCP-1: Monocyte chemoattractant protein-1
IGF-II: Insulin-like growth fator 2
CK-18: Cytokeratin 18
CK18–F: Serum cytokeratin 18 fragments
FLI: The fatty liver index
APRI: Platelet ratio index
FIB-4: Fibrosis Index
NFS: NAFLD fibrosis score
ELF: The Enhanced Liver Fibrosis score
WHTR: Waist-to-height ratio
HA: Hyaluronidase
P3NP: Serum collagen type III N-telopeptide
CHI3L1: Chitinase 3-like protein 1
FAST: FibroScan-AST
3D-MRE: Three-dimensional MR
IV-C: Collagen type IV
A/G: Albumin-globulin ratio
CUS: Conventional ultrasound
HRI: Hepatorenal index
QUS: Quantitative ultrasound
VCTE: Vibration control transient elastography
CAP: Controlled attenuation parameter
LSM: Liver stiffness measurement
MRI: Magnetic resonance images
MRS: Magnetic resonance spectroscopy
cT1: Iron-corrected T1
INR: International normalized ratio
miR-122: MircroRNA-122
miR-192: MircroRNA-192
miR-99a: MircroRNA-99a
MRI-PDFF: Proton density fat fraction
11,12-Dihetre: 11,12-Dihydrox yeicosatrienoic acid
dhk PGD2: 13,14-dihydro-15-keto prostaglandin D_2_
20-COOH AA: 20-carboxy arachidonic acid

## 1 Introduction

Non-alcoholic fatty liver disease (NAFLD) is a complex metabolic condition intimately linked to insulin resistance. There is evidence of potential genetic and environmental risk factors. The following four NAFLD categories can be distinguished: simple steatosis (non-alcoholic fatty liver, NAFL), nonalcoholic steatohepatitis (NASH), NASH-associated cirrhosis, and liver cancer (HCC) [[1], [2], [3]]. In recent years, many nations have come to agree in recent years that the term NAFLD should be changed to metabolic dysfunction associated steatotic liver disease (MASLD) [4,5]. But unlike NAFLD, MASLD involves more than just a vocabulary shift. Changes in terminology, diagnostic standards and clinical characteristics are also involved [6,7]. And the studies and guidelines included in this review are all for NAFLD, so the old nomenclature is still used in this text. Interestingly, 99% of the NAFLD and MASLD populations overlap, according to a review by Terry Cheuk-Fung Yip et al. from the Centre for Healthcare Data Analytics at the Chinese University of Hong Kong on "the continued use of previous NAFLD-related data under the new MASLD definition" [8]. In other words, there is little difference between NAFLD and MASLD, so it stands to reason that the findings of previous NAFLD-related studies are still valid for MASLD. As a result, MASLD can benefit from the current status and potential of non-invasive NAFLD diagnosis discussed in this work.

It is customary to believe that NAFLD is the primary cause of chronic liver disease in China and the rest of the globe [9,10]. The average prevalence of NAFLD is 32.4% worldwide and 34% in Asia, which is comparable in Europe and North America, and the prevalence of NAFLD is increasing at an alarming rate. Of note, the aging of the population, as well as the growing obesity and diabetes epidemic, will probably have a significant impact on its prevalence in the years to come [[11], [12], [13]]. From the available evidence, NAFLD is an independent risk factor for adverse cardiovascular events. The severity of NAFLD is shown to progressively increase the comorbidity or morbidity of adverse cardiovascular events, such as atherosclerosis, ischemic heart disease, hypertension, cerebrovascular disease, and so forth [[14], [15], [16]]. Additionally, NAFLD has been linked to an increased risk of all primary malignancies and certain specific cancers [17]. Since NAFLD is thought to be a "multi-system" illness, multidisciplinary intervention and treatment were needed. The combination of a growing number of NAFLD patients with hepatitis B and other liver or systemic disorders has resulted in a new, emergent public health concern [18,19].

Accurate early-stage diagnosis and management are essential for preventing and treating NAFLD as the illness advances. When alcohol and other potential causes of hepatic steatosis are ruled out, the diagnosis of NAFLD is established with imaging or histological evidence of diffuse hepatocellular steatosis as support. Liver biopsy is the gold standard for distinguishing simple steatosis from NASH and properly evaluating liver fibrosis, although it is an intrusive, expensive, time-consuming diagnostic with sample errors [20].

The NAFLD activity score (NAS), which provided a semi-quantitative method of assessing the pathological alterations and classification and staging of NAFLD, was claimed by the United States NASH Clinical Research Collaboration. The five categories deployed by the NAS for evaluating histological features are steatosis (0–3), inflammation (0–3), hepatocellular injury (0–2), fibrosis (0–4, F0–F4), and other features. The main objective of NAS is to evaluate and summarize the histologically distinct NAFLD lesions as a whole, which cannot be used as a measure of the extent of NAFLD and to differentiate simple steatosis from NASH, as well. Different physicians describe NASH differently under the NAS grading criteria, and different pathologists' observations are not always the same. In addition, the scoring method falls short of accurately capturing all NAFLD characteristics in children. Consequently, it is still challenging to detect the accuracy of NASH [21].

To enhance the repeatability and accuracy of the diagnosis of NASH, the European Fatty Liver Collaboration Group proposed a new scoring system based on essential histological lesions. This included the FLIP algorithm, a diagnostic histology algorithm, and the SAF score, a composite scoring system involving liver steatosis, inflammatory activity, and liver fibrosis. The SAF score, as opposed to NAS, exhibits reduced independent variability, is better at accurately expressing the extent of hepatocyte ballooning, and improves the consistency of pathologists' diagnosis of NASH. However, the association between portal inflammation and the severity of liver injury could not be ascertained by this scoring method because portal inflammation was not taken into account in the FLIP/SAF categorization. Furthermore, scoring thousands of biopsy samples still has the problem of inter-observer error and a large workload [22].

In recent years, there has been an increasing interest in searching for non-invasive markers to evaluate liver fibrosis and differentiate simple steatosis from NASH [23]. Numerous domestic and overseas publications have also investigated the heterogeneity of clinical and pathological characteristics of NAFLD. Thus, this paper will address non-invasive techniques to distinguish simple steatosis from NASH and to evaluate liver fibrosis, which are crucial for the discovery and accurate determination and assessment of prognosis in NAFLD cases based on the study of the differentiation of liver biopsy-confirmed NAFLD cases.

## 2 Recent non-invasive diagnostic methods

### 2.1 Search strategy and selection criteria

We searched PubMed and MEDLINE for studies and reviews published between January 1, 1980 and January 1, 2023 relevant to the scope of the goal with the review “Non-alcoholic Fatty Liver Disease”, “non-alcoholic steatohepatitis”, “NAFLD”, “NASH”, “fatty liver”, “Nonalcoholic Fatty Liver Disease”, “Fatty Liver, Nonalcoholic”, “Fatty Livers, Nonalcoholic”, “Liver, Nonalcoholic Fatty”, “Steatohepatitis, Nonalcoholic”, “non-invasive tests”, “liver fibrosis”, “blood tests”, “diagnosis” and “diagnosis”. Articles were considered regardless of language. We selected references that cross-sectional and cohort studies, whose NAFLD population with biopsy-diagnosed. Most of the articles selected were published within the past 5 years, although we also included highly referenced. We review the most recent non-invasive testing techniques for separating NAFL from NASH (Table 1) and assessing liver fibrosis (Table 2, Table 3).

**Table 1:** Serological markers and other non-invasive tests for diagnose NAFLD. Data from cross-sectional studies.

Markers	N Training set/Validation set	AUC		Diagnostic accuracy
		Training set	Validation set	
Serum based variables				
LDL-TG/LDL-C [34]	44	0.857	NR	cut-off = 0.133
The CK18–F level [39]	246	0.770	NR	cut-off = 260 U/L, PPV = 85.5%, NPV = 52.2%
MRI-PDFF [53]	61	NR	0.929–0.962	
Backscatter coefficient [53]	61	NR	0.811–0.860	
Attenuation coefficient [28]	61	NR	0.779–0.804	
NASHMRI [69]	39/87	0.880	0.830	Training set: cut-off = 0.5, PPV = 80%, NPV = 82% Validation set: cut-off = 0.5, PPV = 71%, NPV = 81%
*ACSL4* cg15536552 [74]	65	0.800	NR	
*CPT1C* cg21604803 [74]	65	0.780	NR	
11,12- DiHETrE [76]	29	1	NR	
Leptin [82]	51/225	0.880	0.830	Training set: cut-off = 9.33 ng/ml, PPV = 58%, NPV = 98%. Validation set: cut-off = 9.33 ng/ml, PPV = 38%, NPV = 97%.
Adiponectin [82]	51/125	0.870	0.630	Training set: cut-off = 7.32 ng/ml PPV = 63%, NPV = 100% Validation set: cut-off = 7.32 ng/ml PPV = 49%, NPV = 71%
*miR-122* [86]	210	NR	0.920	cut-off = 1.261, sensitivity = 92%, specificity = 85%
*miR-99a* [86]	86	NR	0.910	cut-off = 0.460, sensitivity = 94%, specificity = 96%
*miR-34a* [86]	86	NR	0.840	cut-off = 3.070, sensitivity = 84%, specificity = 87%
**Non-invasive tests**				
acNASH index [31]	390/1089	0.818	0.805	Training set: cut-off <4.15, NPV = 83% cut-off >7.73, PPV = 85% Validation set: cut-off <4.15, NPV = 93% cut-off >7.73, PPV = 73%
A panel including dhk PGD2 and 20-COOH AA [76]	29	1	NR	NR
A panel of TGs [77]	467/192	0.950	0.790 ± 0.040	Training set: PPV = 89% NPV = 90% Validation set: cut-off = 0.5, PPV = 81%, NPV = 69%.
EML [81]	144/50	NR	NR	NAFLD identification accuracy: 96.8% NASH identification accuracy: 81.3% NAFL identification accuracy: 94.0%
The score combines with CK18-Asp396 fragment level [84]	198	0.810	NR	NR
NIS4 test [44]	239/702	0.800	0.800	cut-off ＞ 0.36, sensitivity: 81.5%, specificity: 63.0%, NPV: 77.9% cut-off ＜ 0.63: specificity: 87.1%, sensitivity: 50.7%, PPV: 79.2%

AUC: AUROC, area under the receiver operating characteristic; PPV: positive predictive value; NPV: negative predictive value; LDL-TG/LDL-C: Low-density lipoprotein triglyceride/Low-density lipoprotein cholesterol; CK18–F: Serum cytokeratin 18 fragments; MRI-PDFF: Proton density fat fraction; NASHMRI: the diagnostic accuracy of NAFLD detection; 11,12-Dihetre: 11,12-Dihydrox- yeicosatrienoic acid; *miR-122*: mircroRNA-122; *miR-99a*: mircroRNA-99a; *miR-34a*: mircroRNA-34a; acNASH index: acNASH = AST (U/L)/SCr (mmol/L) * 10; A panel of TGs: combined with the BMI and a classification algorithm; EML: an ensemble machine learning model was built (comprehending 10 different mathematical models); The score combines with CK18-Asp396: the score is a combination score of miR-122, -192 and −21; NIS4: The derived NIS4 algorithm comprised four independent NASH-associated biomarkers (miR-34a-5p, alpha-2 macroglobulin, YKL-40, and glycated haemoglobin).

**Table 2:** Serological markers and other non-invasive tests for NAFLD-Related Fibrosis. Data from cross-sectional studies.

markers	N Training set/Validation set	AUC		Diagnostic accuracy
		Training set	Validation set	
3D-MRE [66]	100	NR	0.981	at 60 Hz: AUC = 0.927 Cut-off = 3.40 kPa, PPV = 72.2%, NPV = 97.5% at 40 Hz: AUC = 0.981 Cut-off = 2.43 kPa, PPV = 72.2 %, NPV = 1.0%
2D-MRE [66]	100	NR	0.921	at 60 Hz: Cut-off = 3.80 kPa, PPV = 81.2%, NPV = 97.6%
Fibro MRI [69]	39/87	0.940	0.850	Training set: cut-off = 0.5, PPV = 77%, NPV = 86% Validation set: cut-off = 0.5, PPV = 67%, NPV = 87%.
MRE [64]	104	NR	0.820	F2: AUC = 0.89, F3: AUC = 0.87, F4: AUC = 0.87
RP11‐128N14.5 lncRNA [83]	88/50	0.706	0.694	Training set: sensitivity = 73.7%, specificity = 70.4% Validation set: sensitivity = 78.3%, specificity = 63%,
BNIP3L mRNAs [83]	88/50	0.676	0.686	Training set: sensitivity = 57.9%, specificity = 81.5% Validation set: sensitivity = 51.9%, specificity = 91.3%,
Complement component C7 [88]	19	1	NR	NR
α-2-macroglobulin [88]	19	0.870	NR	NR
Complement component C8 γ chain [88]	19	0.800	NR	NR
Fibulin-1 [88]	19	0.790	NR	NR
α-1-antichymotrypsin [88]	19	0.800	NR	NR
the FM-fibro LSM index [60]	249	0.943	0.941	cut-off = 7.201, PPV = 73.5%, NPV = 94.6%
top 10 metabolite panel [78]	156/59	0.940	0.840	Sensitivity = 90%, specificity = 79%
IGF-1 + ferritin + INR [82]	225	NR	0.810	PPV = 98%, NPV = 71%
TGFB2/TGFB2‐OT1 Test [83]	88/50	0.797	0.786	Training set: sensitivity = 65%, specificity = 81.3% Validation set: sensitivity = 62.5%, specificity = 94.4%
TGFB2/TGFB2‐OT1 + FIB‐4 [83]	88/50	0.891	0.889	Training set: sensitivity = 80%, specificity = 87.5% Validation set: sensitivity = 87.5%, specificity = 83.3%
7 eicosanoids panel [35]	426	0.740	NR	NR

3D-MRE: Three-dimensional MRE; 2D-MRE: two-dimensional MRE; Fibro MRI: the diagnostic accuracy of significant fibrosis prediction; MRE; magnetic resonance elastography; the FM-fibro LSM index: combination of the FM-fibro index and LSM; top 10 metabolite panel: the top 10 serum metabolites including 5alpha-androstan-3beta monosulfate, pregnanediol-3-glucuronide, androsterone sulfate, epiandrosterone sulfate, palmitoleate, dehydroisoandrosterone sulfate, 5alpha-androstan-3beta disulfate, glycocholate, taurine and fucose; 7 eicosanoids panel: 5-HETE, 7,17-DHDPA, adrenic acid, arachidonic acid, eicosapentaenoic acid, 16-HDOHE, and 9-HODE.

**Table 3:** Serological markers and other non-invasive tests for NAFLD-Related Fibrosis. Data from cohort studies.

markers	N Training set/Validation set	study design	AUC		Diagnostic accuracy
			Training set	Validation set	
ELF test [48]	371	a multicenter cohort study	0.802	NR	F0 VS F1-4: AUC = 0.825 F0-1 VS F2-4: AUC = 0.817 F0-2 VS F3-4： AUC = 0.802 F0-3 VS F4： AUC = 0.812 F ≥ 3: AUC = 0.802
nomogram test [38]	207	a prospective cohort study	0.829	NR	F ≥ 2 AUC = 0.829 cut-off = 50, PPV = 79.1%, NPV = 72.5%
LRM [49]	278/275	a centre prospective derivation	0.764	0.786	Training set: PPV = 66.4%, NPV = 66.4% Validatio set: PPV = 71.1%, NPV = 85.4%
MLA [49]	278/275	a centre prospective derivation	0.902	0.893	F ≥ 2： Training set: AUC = 0.902 Validation set: AUC = 0.893 F ≥ 3: AUC = 0.996 F4: AUC = 0.989
FAST [62]	350/1026	a prospective study	0.800	0.85	Cut-off ≤0.35, NPV = 94.0% Cut-off ≥0.67, PPV = 69.0%
gadoxetate-based methods [65]	90	a prospective study	NR	0.68–0.81	sensitivity = 71%
MEFIB [68]	234/314	a prospective study	0.860	0.899	Cut- off: MRE = 3.3 kPa, FIB-4 = 1.6 F = 2: Training set: PPV = 91.2%, NPV = 92.8% Validation set: PPV = 95.6%, NPV = 85.6% F2 and F3: AUC = 0.880 PPV = 93.0%
MAST [73]	103/244	a retrospective study	0.858	0.929	Cut-off = 0.165 Training set: sensitivity = 94.40%, NPV = 98.40% Validation set: sensitivity = 89.30%, NPV = 98.10% Cut-off = 0.242 Training set: specificity = 89.40%, NPV = 91.60% Validation set: specificity = 90.30%, NPV = 96.50%
Four proteins panel [87]	113	a retrospective study	0.740	NR	
12 proteins model [87]	113	a retrospective study	0.830	NR	

ELF test: The enhanced liver fibrosis test; nomogram test: Variables included waist-to-height ratio, hyaluronic acid, procollagen–III–peptide, chitinase-3-like protein 1, and cytokeratine-18 neoepitope M65; LRM: a logistic regression model, these predictors were BMI, serum procollagen type III (PC--III), type IV collagen (IV--C), AST and A/G ratio. LRM formula (0.00265 × AST + 0.00426 × PC--III + 0.00669 × IV--C + 0.01893 × BMI − 0.53322 × A/G); MLA: the machine learning algorithm, included the following five variables (BMI, PC--III, IV--C, AST and A/G ratio),The relative importance of the above-mentioned variables in MLA were 100% for PC--III, 87% for IV--C, 78% for BMI, 68% for AST and 60% for A/G ratio; FAST: FibroScan-AST; MEFIB index: combining FIB-4 as a serum-based marker and MRE; MAST: The MRI-aspartate aminotransferase score, MAST = −12.17 + 7.07 log MRE + 0.037 PDFF + 3.55 log AST; Four proteins: serum amyloid P [SAP], fibrinogen, olfactomedin, and SHBG; 12 proteins model: consists of LTBP4, IGF-1, VCAM1, IL1SRI, IL18Bpa, TSP2, collectin kidney 1, SHBG, TCCR, LIFsR, FBLN3, and PLXB2.

### 2.2 Guidelines for non-invasive assessments

To assess the risk of NAFLD complicated with liver fibrosis, the Liver Fibrosis Prediction Calculation is advised by both the European Association for the Study of the Liver (EASL) and the American Association for the Study of Liver Diseases (AASLD). The preferred non-invasive test (NIT) is the Fibrosis-4 Index (FIB-4). Both guidelines concur that FIB-4 is not a direct marker of liver fibrosis, and doctors shouldn't base their decisions only on it or comparable indirect non-invasive testing. According to AASLD, patients with high or indeterminate FIB-4 results ought to have additional testing done, such as transient elastography (LSM) or the Enhanced Liver Fibrosis (ELF) test [[24], [25], [26]]. As compared to utilizing a single non-invasive test (NIT), it has been shown that combining two NITs, such as FIB-4 and Vibration control transient elastography (VCTE) or VCTE and proprietary serological tests, can enhance diagnostic accuracy [27]. The Asia-Pacific guidelines also agree that combining imaging equipment with serum testing may yield more accurate results than using either technique alone [28]. However, they do not specify which non-invasive test is better.

EASL recommends using ALT, AST, and platelet counts as part of routine investigations in primary care for patients with suspected liver disease to calculate a simple, non-invasive score. However, it is worth noting that the National Institute for Health and Care Excellence (NICE) discourages routine liver blood testing to rule out NAFLD. Compared to AASLD and EASL, in patients with NAFLD, NICE advises considering the preferred use of the ELF test for identifying advanced liver fibrosis [26,29]. Since non-invasive diagnostic techniques are still limited and lack the necessary clinical evidence for routine clinical practice, the guidelines advocate liver biopsy for the diagnosis of NAFL and NASH [24,30].

### 2.3 Serological markers

#### 2.3.1 Single serological markers

Throughout the last decade, a number of non-invasive serum biomarkers have been demonstrated to differentiate between NAFLD. Multiple single serological indicators are significant in the diagnosis of NAFLD. In one study of 1479 patients, there were statistically significant differences between the pathological characteristics of NAFLD and age, sex, body mass index (BMI), aspartate aminotransferase (AST), alanine aminotransferase (ALT), ALT/AST ratio, serum creatinine (SCR), estimated glomerular filtration rate (e-GFR), total cholesterol, and uric acid (UA) [31]. Despite the fact that NAFLD patients usually have high blood ALT levels, these levels are normally not employed as a diagnostic sign [32]. Moreover, studies have connected anomalies in lipid metabolism to a higher risk of NAFLD [33]. For instance, the ratio of low-density lipoprotein triglyceride to low-density lipoprotein cholesterol (LDL-TG/LDL-C) was a non-invasive and promising way to distinguish NASH from NAFL. Particularly, LDL-TG/LDL-C levels were associated with specific inflammation, ballooning, and fibrosis intensity [34]. In research with 31 healthy living liver donors as healthy controls, there were substantial variations in the plasma phospholipid profile (mainly docosahexaenoic acid, n-6 and n-3 fatty acids, etc.) between 26 patients with NAFL and 20 with NASH and normal individuals [33]. In 426 patients with biopsy-confirmed NAFLD, seven eicosanoids were tentatively shown to be strongly linked with the fibrosis stage. It is believed that patients with NAFLD can employ plasma eicosanoid compounds as a non-invasive indicator of liver fibrosis and improvement in hepatic fibrosis. Notably, further research is required to validate the diagnostic accuracy of detecting liver fibrosis improvement in NAFLD [35].

Progression of NAFLD tends to be uncertain, and clinical manifestations, laboratory tests and so on, may be inconsistent with pathological changes. Accordingly, reliable, non-invasive methods are needed to monitor the severity and progression of NAFLD [20]. A recent large sample study shows that activated plasminogen activator inhibitor 1 (APAI1) is interrelated with NASH and associated with significant fibrosis (relative to mild/no fibrosis), including high levels of interleukins-8 (*IL-8*), monocyte chemoattractant protein-1 (MCP-1), resistin, soluble IL-1 receptor, soluble IL-2 receptor alpha, tumor necrosis factor-alpha and low levels of insulin-like growth factor 2 (*IGF-II*), which may have potential value for non-invasive stratification of patients with NAFLD and identification of therapeutic intervention targets. And since this study was primarily cross-sectional, more longitudinal research would be beneficial [36].

Main intermediate filament protein in hepatocytes is referred to as cytokeratin 18 (CK-18), and the majority of such insoluble intracellular proteins are digested by caspase and broken into the M30 and M65 fragments that are released externally during hepatocyte apoptosis [37]. Consequently, several investigations revealed that there was a significant difference between the pathological features of NAFLD patients and the serum levels of cytokeratin 18-M65 (CK18-M65), which allowed for the identification of patients with NAFLD and early fibrosis stages (stages 1 and 2), as well as the progression of fibrosis [38].

Due to the heterogeneity between CK18 and pathological features of NAFLD, a study including 246 patients histologically diagnosed with NASH (n = 185) or nonalcoholic fatty liver (n = 61) found that serum CK18 fragments (CK18–F) levels did not differ among fibrosis stages but did significantly differ among hepatocyte ballooning grades. Even when FIB-4 is low, NASH can be reliably and non-invasively predicted by combining the CK18 level and FIB-4 index [39]. In contrast, other studies have demonstrated that apoptotic biomarkers, including CK18-M30, CK18-M65, and their combination, cannot differentiate between NASH and simple steatosis [40].

#### 2.3.2 Combined use of non-invasive serological markers

Compared to single biomarkers, combination of non-invasive serum biomarkers can further improve diagnostic performance [41]. A more recent large meta-analysis evaluated the six most prevalent and used co-scores, involving the fatty liver index (FLI), AST, platelet ratio index (APRI), FIB-4 index, AST/ALT 4 ratio, Bard score, and NAFLD fibrosis score (NFS), besides quantitative analysis of it, which testified that FLI of NAFLD and APRI of NASH are the combined scores with the highest diagnostic and prognostic value [42]. In the clinical setting, AST, APRI, NFS, and FIB-4 are the ones that are utilized the most. While their effectiveness in ruling out severe fibrosis is commendable, none of them have demonstrated sufficient accuracy in differentiating between low-level liver fibrosis and the presence of liver fibrosis [38,43]. Consequently, further investigation and assessment of novel scores will be helpful in developing non-invasive techniques that can precisely distinguish NAFL from NASH, measure liver fibrosis, and determine prognosis. Recently, a new blood-based diagnostic way called NIS4 (specificity: 87.1%), which was significantly superior to others in identifying elevated-risk NASH, such as FIB-4, NFS, APRI, and BARD (all *P* < 0.05), and is an effective method to spot high-risk NASH (NAS ≥4 and fibrosis stage ≥2) in patients with metabolic risk factors like type 2 diabetes, obesity, dyslipidemia, hypertension and no alternative causes of chronic liver disease or fatty degeneration [44].

It has been shown in one of the finest-ever surveys of individuals with biopsy-confirmed NAFLD that the timing of the development of severe liver disease can be explained in terms of the stage of fibrosis. Advanced fibrosis was associated with liver-specific morbidity and overall mortality [45]. The ELF score, which can be paired with the FIB-4 score to raise diagnostic accuracy, is a current algorithm for identifying terminal liver fibrosis proposed by the EASL [46,47]. In addition, a Japanese study evaluating the diagnostic efficacy of ELF found that it had a similar predictive ability to other indications for early fibrosis in NAFLD. When different characteristics were incorporated, the ELF's sensitivity and specificity for predicting advanced fibrosis (F ≥ 3) increased to 91.1%, 50.8%, 38.5%, and 92.8%, respectively, which made it more accurate for identifying F3 or F4 [48].

ZHOU et al. developed a new nomograph-based non-invasive model by combining waist-to-height ratio (WHTR), hyaluronidase (HA), serum collagen type III N-telopeptide (P3NP), chitinase 3-like protein 1 (CHI3L1), and CK-18 M65, which was more accurate in diagnosis than APRI, NFS, FIB-4, and BARD (AUC = 0.829, 95% CI: 0.755–0.904), also can as a non-invasive treatment for individualized prediction of F2 and assessment of the risk of significant fibrosis in patients with NAFLD [38]. Afterward, a study in 2021 created an MLA model with the following five variables: BMI, serum procollagen type III (PC-III), collagen type IV (IV–C), AST, and Albumin-globulin (A/G) ratio. MLA had the highest diagnostic accuracy compared with other diagnostic models (AUC = 0.902, 95% CI: 0.869–0.904). Additionally, MLA showed excellent performance in predicting advanced fibrosis (AUC = 0.996, 95% CI: 0.967–0.998), similar to predicting the presence of cirrhosis (AUC = 0.989, 95% CI: 0.977–0.996). However, it is noteworthy that since this discussed cohort mainly included patients with early liver fibrosis, five and a few patients with advanced fibrosis (F ≥ 3), the MLA algorithm might, therefore, be valuable in predicting fibrosis (F ≥ 2) tools [49].

### 2.4 Imaging diagnosis

#### 2.4.1 Based on ultrasonic imaging technology

The previously mentioned serum biomarkers and their derived scoring methods have been certified as moderately valuable and these can be investigated further in conjunction with the development of contemporary non-invasive methods in other contexts. Future modifications to surveillance recommendations may result from variations in results based on hematological parameters and advancements in imaging modalities. Additionally, several approaches have found imaging techniques combine serum biomarkers as a potent substitute for biopsy in the assessment of NAFLD.

When fatty liver is suspected, ultrasound imaging techniques tend to serve as the first line of defense, although conventional ultrasound (CUS) has some drawbacks, including excessive interobserver variability and the inability to determine mild steatosis in NAFLD, CUS has been the most widely used radiograph to assess steatosis. Previously, the hepatorenal index (HRI) was proposed as a reliable quantitative method for identifying hepatic steatosis. Unfortunately, HRI has limitations for the diagnosis of some patients with renal disease or anatomical changes related to physiological processes, and it is ineffective at differentiating between liver fatty and mild steatosis [[50], [51], [52]]. The objective evaluation of liver steatosis in patients has a lot of promise for quantitative ultrasound (QUS) technology. Two fundamental liver tissue metrics, the attenuation coefficient and the backscatter coefficient, have been examined in a study and were thought to have better inter-observer consistency and be more accurate than CUS at detecting shifts in liver fatty composition [53].

It is well known that VCTE is one of the most widely used in elastography, and recently, measuring the ultrasonic attenuation of echoes (called controlled attenuation parameter, CAP) supplements the ability to quantify hepatic steatosis [54]. With the increase of hepatic tissue fatty lesions and inflammation, CAP value gradually increases, which is fairly accurate for detecting severe hepatic steatosis. Its veracity is yet affected by obesity, and the accuracy of QUS may be less affected by obesity than CAP [53,55]. Fortunately, VCTE can now be accurately conducted in obese populations thanks to the invention of the XL probe, which is frequently utilized in patients with a BMI >30 kg/m^2^ [56,57]. The higher the degree of liver fibrosis in NAFLD patients, the LSM values. Therefore, liver fibrosis can be evaluated by the LSM value of VCTE, but the applicability (90% of NAFLD patients) and accuracy (AUC = 0.891) of the LSM in NAFLD patients are slightly limited. However, a more considerable skin capsule distance affects the diagnostic performance of CAP and LSM [58], and VCTE is less accurate than CAP in distinguishing between low fibrosis, severe steatosis grade, or the presence or absence of NASH [59]. To further promote its detection accuracy and applicability, studies have combined LSM with various biomarkers (AUC = 0.937), particularly for predicting advanced fibrosis, with the FM-fibro LSM index (combination of the FM-fibro index and LSM) showing the highest AUC among the various combinations of biomarkers and LSM [60].

FAST (FibroScan - AST), a novel, straightforward, non-proprietary score combining LSM, CAP, and AST for identifying individuals with progressive NASH, has been validated in large-scale worldwide cohorts. FAST score is sensitive to each histological feature and can non-invasively identify patients at risk for progressive NASH. This decreases the need for unnecessary liver biopsies, even though the algorithm discriminates differently across nations [61]. A search for external validation of the FAST algorithm is noteworthy as it confirms the discrepancy and the strong association between the FAST algorithm and the fibrosis phase of NAFLD [62].

#### 2.4.2 Imaging techniques based on MRI

The most widely used MRI-based (magnetic resonance images-based) elastography technique is magnetic resonance elastography (MRE), which was more accurate than VCTE in identifying liver fibrosis (F ≥ 1) using biopsy analysis as a standard. MRE has a favorable correlation with fibrosis staging (r = 0.66, P = 0.001) and is the best predictor of advanced fibrosis (F2–F4) [63,64]. Additionally, MRE has a particularly elevated diagnostic accuracy for advanced fibrosis, distinguishing fibrosis in stages F0, F2 and F3. Later, following the development of two-dimensional MRE, three-dimensional (3D) -MRE, a more advanced technique, was created. 3D-MRE may enhance the diagnostic performance of MRE for advanced fibrosis, especially since the shear wave frequency is 40 Hz [[65], [66], [67]]. Recently, there is a new study compared the diagnostic accuracy of MEFIB (the combination of MRE and FIB-4) and FAST for diagnosing significant fibrosis (F = 2) and found the former to be more effective than the latter and was incorporated into the 2023 AASLD Practice Guidance on the clinical assessment and management of nonalcoholic fatty liver disease [24,68].

One study evaluated tools based on optical analysis of liver MRI: The diagnostic accuracy of NAFLD detection (NASHMRI) was 0.83 (95% CI: 0.73–0.93), and the diagnostic accuracy of significant fibrosis prediction (Fibro MRI) was 0.85 (95% CI: 0.77–0.94). Thus MRI optical analysis may have significant potential as a non-invasive imaging biomarker but may be impractical for routine clinical use because of the large number of patients affected [69].

Based on MRI and magnetic resonance spectroscopy (MRS), 1H proton density fat fraction (PDFF) can be easily obtained and gradually applied to clinical diagnosis. A follow-up study demonstrated that liver fat content measured by MRI-PDFF, the best non-invasive test for liver fat, was associated with fibrosis progression and was more accurately detected than CAP. This promising mode requires further external validation. Additionally, iron-corrected T1 (cT1) may also be a promising quantitative non-invasive imaging biomarker, which is not only able to detect liver fat alone, but also has advantages in detecting inflammation and fibrosis [64,[70], [71], [72]]. More recently, FORSGREN et al. certified that gadoxetine-based MRI is sufficiently responsive to detect low-function loss associated with fibrosis, and established that the gadoxetine-enhanced MRI algorithm outperforms the best serum fibrosis algorithm in distinguishing low-to-advanced stages of fibrosis [65]. A recent paper derived and validated a score called MRI-aspartate aminotransferase (MAST) score that can stratify risk in patients with NAFLD by assessing steatosis and fibrosis separately. It is combines MRI-MRE and MRI-PDFF with AST to identify fibrosis with excellent specificity. This score narrows down patients in the gray area and has balanced accuracy. However, this is a retrospective study and the histological scores may vary, so further validation may be needed [73].

## 3 Non-invasive diagnostic methods based on multi-omics

Recently, an innovative method to detect NAFL and NASH, assess liver fibrosis, and determine prognosis has been discovering a set of chemicals strongly related to illness using omics techniques (genomics, lipidomics, metabolomics, transcriptomics, proteomics) [43].

### 3.1 Based on genomics

Genome-wide DNA methylation profile analysis was performed on peripheral blood leukocytes in a cohort of NAFLD patients and normal controls with NAFLD, confirming that hypomethylation of *ACSL4* (CG15536552) and *CPT1C* (CG21604803) can increase the risk and susceptibility of NAFLD and hepatic steatosis (*ACSL4* cg15536552, OR: 10.50, 95% CI: 1.70–64.99, *P* = 0.014; *CPT1C* cg21604803, OR: 7.67, 95% CI: 2.14–27.49, *P* = 0.001). Therefore, peripheral blood leukocyte DNA methylation profile may serve as a non-invasive biomarker for pathological assessment of NAFLD [74].

### 3.2 Based on lipid and metabolomics

Several research projects have concentrated on tracking the course of NAFLD and refining the reliability of stratified NAFLD diagnosis. A study presented 20 comparable plasma metabolite panels that could unmistakably distinguish NASH from fatty change, and lipid and water metabolites may be considered indicators for disease progression in NAFLD [75]. Regarding dictation NASH and improving the diagnostic veracity of fibrosis, 11,12 - Dihydrox - yeicosatrienoic acid (11,12-Dihetre) and a panel including 13,14-dihydro-15-keto prostaglandin D_2_ (dhk PGD2) and 20-carboxy arachidonic acid (20-COOH AA) have long been affirmed the main candidate biomarkers for non-invasive diagnosis of NASH, and their AUC is 1. Moreover, PGE2, dhk PGD2, Tetranor 12 - HETE, 15 - HETE, 14,15 - diHETrE, 9 - oxoODE, 12,13 - EpOME may provide novel biomarkers candidate for non-invasive detection of NASH (AUC = 0.73–0.96). Additional research is required to evaluate the accuracy and reliability of these putative biomarkers due to the modest size of the cross-sectional investigation and the absence of a large validation cohort [76]. Whereafter, Mayo et al. developed an entirely novel diagnostic algorithm based on a sizable cohort of 20 serum triglycerides from biopsy-confirmed patients that distinguished NASH from NAFL with an AUC of 0.79 and had a sensitivity of 70% and specificity of 81%; notwithstanding, the algorithm's accuracy was impacted by the presence or absence of type 2 diabetes (AUC = 0.69) [77]. Since then, CauSSy et al. have discovered that ten related metabolites can be used to predict the presence of advanced fibrosis in the study using non-targeted metabolite profiles. By using validation cohorts with biopsy-proven NAFLD, they have also determined that the diagnostic accuracy of this panel of 10 related metabolites (AUC = 0.94) was superior to FIB-4 and NAFLD fibrosis scores [78].

In non-obese and obese NAFLD patients, altered serological levels of diacylglycerol, triacylglycerol, and saturated sphingomyelin differ from the occurrence of NAFLD and histological severity [79]. Subsequent studies using metabolome analysis have identified several candidate metabolites (such as phosphatidylcholine) for distinguishing NAFL from NASH. They are also used in the clinic to spot the recurrence of NAFLD after liver transplantation and to distinct NAFL from advanced nonalcoholic steatohepatitis, besides needing larger sample sizes for further studies [80]. In the same year, a non-targeted metabonomics study revealed that serum metabonomics features could help validate NAFLD and distinguish fibrosis stages, confirming that isoleucine, valine, and asparagine are strongly associated with NAFLD disease progression and may be potential biomarkers for NAFLD [81].

Ultimately, a study comparing obese patients with biopsy-proven NAFLD to healthy controls concluded that blood leptin may be used to detect NAFLD. The stratified diagnosis of NAFLD may also benefit from the use of adiponectin in conjunction with certain lipids as a new detection strategy, which may further enhance the detection effect (AUC = 0.80, *P* ≤ 0.01) IGF-1, ferritin, and the international normalized ratio (INR) can also be considered as a useful biomarker panel to detect advanced fibrosis (AUC = 0.81, *P* ≤ 0.01) because they are simple to employ in clinical settings and more accurate than traditional methods. Hence, serum metabolomics will offer fresh opportunities to strengthen the non-invasive biomarker panel [82].

### 3.3 Based on transcriptomics

It has been reported that coding/non-coding RNA can be used as a non-invasive biomarker for diagnosing NAFLD and evaluating the severity of fibrosis, which can be combined with clinical data to better non-invasive screening of NAFLD patients [83]. At the onset of steatosis and the progression of NASH, levels of the CK18-ASP396 segment, like mircroRNA-122 (*miR-122*) and mircroRNA-192 (*miR-192*), increased in a manner. It positively correlated with corresponding microRNAs (miRNAs) levels that control gene expression. Early detection may improve current diagnostic techniques by using miRNA as a biomarker, and a composite score incorporating *miR-122, -192, and -21*, previously demonstrated to be linked with NAFLD. Combining the score with the CK18-Asp396 fragment level allowed for the best observations (AUC = 0.83 95% CI: 0.754–0.908) [84]. The strong diagnostic accuracy of *miR-122* and the potential utility of circulating miRNAs in diagnosing NAFLD were further corroborated by a meta-analysis of 1408 individuals with NAFLD confirmed by biopsy in 2019 [85]. In the same year, 210 patients with NAFLD participated in a case-control study, thereby supporting *miRNA-122*'s role as a reliable indicator of the disease. Moreover, the levels of *miRNA-122* and *miRNA-34a* were significantly positively correlated with all histology tests, and the down-regulation of the *miRNA-99a* level was a useful predictor of NASH progression with a high sensitivity of 94% [86].

### 3.4 Non-invasive diagnostic methods based on proteomics

In the discovery cohort (n = 113), using the Somascan proteomics platform quantified 1305 serum proteins and found that 97 proteins with cross-cultural biological functions can distinguish between the late stage (stage 3–4) and early stage (stage 0–2) of fibrosis in NASH patients, but that no protein can distinguish between fibrosis stage 2 from stages 0–1, 3, and 4. So, further analysis will benefit these biomarkers' clinical applicability [87]. For the first time in 2020, complement component C7, complement component C8 γ chain, and fibulin-1 were discovered to be significantly associated with liver stiffness, highlighting complement C7 as a potential biomarker (AUC = 1) to identify NASH patients with significant/advanced liver fibrosis (F2 – F4). However, more analysis with larger samples is necessary [88].

## 4 Conclusion

As seen from the discussion above, there are still no accurate non-invasive serum biomarkers and algorithms to measure steatosis and fibrosis in NAFLD, accurately distinguish NAFL from NASH, assess liver fibrosis, and forecast prognosis. Nonetheless, several serum biomarkers and algorithms, such as MRI-PDFF, *miR-122*, *miR-99a*, FM-Fibro scan LSM index, and complement component C7 (AUC ≥0.90), have demonstrated a great deal of potential to supplant biopsy. Larger sample sizes and longitudinal studies are therefore required for the long-term validation of these biomarkers and algorithms. In particular, non-invasive, rigorous, and repeatable alternatives can be found to track the disease staging over time and different stages of NAFLD (NAFL, NASH, or liver fibrosis).

Secondly, some studies have found biomarkers and algorithms that can accurately distinguish advanced fibrosis from other types of fibrosis, like MLA (F≥3: AUC = 0.996), while few studies precisely identify early fibrosis, especially F2 stage fibrosis, this may be a direction for future research. In the end, with the development of omics technology, the search for biomarkers is not limited to serum biomarkers. It is believed that additional biomarkers can be found in genes, proteins, metabolism and other aspects that can accurately identify NAFL and NASH, evaluate fibrosis, and track the development of NAFLD illness. In summary, we hope that this review will assist us identify alternative non-invasive strategies for early, successful NAFLD intervention and halting disease progression.

## Ethical approval

Ethical approval and consent of patients to participate is not applicable to our study.

## Data availability statement

Data for the review paper are all included to the document and hence no other data available.

## CRediT authorship contribution statement

**Jia-Lan Wang:** Writing – original draft, Writing – review & editing. **Su-Wen Jiang:** Writing – original draft, Writing – review & editing. **Ai-Rong Hu:** Conceptualization, Formal analysis, Funding acquisition, Methodology, Resources, Supervision, Validation, Writing – review & editing. **Ai-Wu Zhou:** Writing – review & editing. **Ting Hu:** Writing – review & editing. **Hong-Shan Li:** Writing – review & editing. **Ying Fan:** Writing – review & editing. **Ken Lin:** Writing – review & editing.

## Declaration of competing interest

The authors declare that they have no known competing financial interests or personal relationships that could have appeared to influence the work reported in this paper.

## References

[1] Paternostro R., Trauner M. (2022). Current treatment of non-alcoholic fatty liver disease. J. Intern. Med..

[2] Cariou B. (2021). Nonalcoholic fatty liver disease as a metabolic disease in humans: a literature review. Diabetes Obes Metab.

[3] Diehl A.M., Day C. (2017). Cause, Pathogenesis, and treatment of nonalcoholic steatohepatitis. N. Engl. J. Med..

[4] Rinella M.E. (2023). A multi-society Delphi consensus statement on new fatty liver disease nomenclature. J. Hepatol..

[5] Eslam M. (2020). MAFLD: a consensus-Driven proposed nomenclature for metabolic associated fatty liver disease. Gastroenterology.

[6] Gofton C. (2023). MAFLD: How is it different from NAFLD?. Clin. Mol. Hepatol..

[7] Wai-Sun Wong V., Kanwal F. (2021). On the proposed definition of metabolic-associated fatty liver disease. Clin. Gastroenterol. Hepatol..

[8] Song S.J. (2024). Can we use old NAFLD data under the new MASLD definition?. J. Hepatol..

[9] Cheemerla S., Balakrishnan M. (2021). Global Epidemiology of chronic liver disease. Clin. Liver Dis..

[10] Younossi Z.M. (2019). Burden of illness and Economic model for patients with nonalcoholic steatohepatitis in the United States. Hepatology.

[11] Le M.H. (2019). Global NAFLD prevalence: a systematic review and meta-analysis. Clin. Gastroenterol. Hepatol..

[12] Riazi K. (2022). The prevalence and incidence of NAFLD worldwide: a systematic review and meta-analysis. Lancet Gastroenterol Hepatol.

[13] Le M.H. (2023). Global incidence of non-alcoholic fatty liver disease: a systematic review and meta-analysis of 63 studies and 1,201,807 persons. J. Hepatol..

[14] Xu J. (2021). Severity of nonalcoholic fatty liver disease and risk of future ischemic Stroke events. Stroke.

[15] Simon T.G. (2022). Non-alcoholic fatty liver disease and incident major adverse cardiovascular events: results from a nationwide histology cohort. Gut.

[16] Alon L. (2022). Risk of cardiovascular events in patients with non-alcoholic fatty liver disease: a systematic review and meta-analysis. Eur J Prev Cardiol.

[17] Taylor A. (2022). Metabolic dysfunction-related liver disease as a risk factor for cancer. BMJ Open Gastroenterol.

[18] Mantovani A. (2022). Risk of heart failure in patients with nonalcoholic fatty liver disease: JACC review topic of the week. J. Am. Coll. Cardiol..

[19] (2018). National workshop on fatty liver and alcoholic liver disease, Chinese society of hepatology, Chinese medical AssociationFatty liver expert committee, Chinese medical doctor association. Guidelines of prevention and treatment for nonalcoholic fatty liver disease: a 2018 update. Chin. J. Hepatol..

[20] Reinson T. (2022). Noninvasive serum biomarkers for liver fibrosis in NAFLD: current and future. Clin. Mol. Hepatol..

[21] Kleiner D.E. (2005). Design and validation of a histological scoring system for nonalcoholic fatty liver disease. Hepatology.

[22] Nascimbeni F. (2020). Clinical validation of the FLIP algorithm and the SAF score in patients with non-alcoholic fatty liver disease. J. Hepatol..

[23] Di Mauro S. (2021). Clinical and molecular biomarkers for diagnosis and staging of NAFLD. Int. J. Mol. Sci..

[24] Rinella M.E. (2023). AASLD Practice Guidance on the clinical assessment and management of nonalcoholic fatty liver disease. Hepatology.

[25] Cusi K. (2022). American association of clinical endocrinology clinical practice guideline for the diagnosis and management of nonalcoholic fatty liver disease in primary care and endocrinology clinical settings: Co-sponsored by the American association for the study of liver diseases (AASLD). Endocr. Pract..

[26] (2021). EASL Clinical Practice Guidelines on non-invasive tests for evaluation of liver disease severity and prognosis - 2021 update. J. Hepatol..

[27] Canivet C.M. (2023). Validation of the new 2021 EASL algorithm for the noninvasive diagnosis of advanced fibrosis in NAFLD. Hepatology.

[28] Chitturi S. (2018). The asia-pacific working party on non-alcoholic fatty liver disease guidelines 2017-Part 2: management and special groups. J. Gastroenterol. Hepatol..

[29] National Guideline C. (2016). Non-Alcoholic Fatty Liver Disease: Assessment and Management.

[30] Kang S.H. (2021). KASL clinical practice guidelines: management of nonalcoholic fatty liver disease. Clin. Mol. Hepatol..

[31] Wu X.X. (2021). acNASH index to diagnose nonalcoholic steatohepatitis: a prospective derivation and global validation study. EClinicalMedicine.

[32] Chalasani N. (2018). The diagnosis and management of nonalcoholic fatty liver disease: practice guidance from the American Association for the Study of Liver Diseases. Hepatology.

[33] Ma D.W. (2016). Plasma phospholipids and fatty acid composition differ between liver biopsy-proven nonalcoholic fatty liver disease and healthy subjects. Nutr. Diabetes.

[34] Fujii Y. (2020). Low-density lipoprotein (LDL)-Triglyceride and its ratio to LDL-cholesterol as diagnostic biomarkers for nonalcoholic steatohepatitis. J Appl Lab Med.

[35] Caussy C. (2020). Plasma eicosanoids as noninvasive biomarkers of liver fibrosis in patients with nonalcoholic steatohepatitis. Therap Adv Gastroenterol.

[36] Ajmera V. (2017). Novel plasma biomarkers associated with liver disease severity in adults with nonalcoholic fatty liver disease. Hepatology.

[37] He L. (2017). Diagnostic value of CK-18, FGF-21, and related biomarker panel in nonalcoholic fatty liver disease: a systematic review and meta-analysis. BioMed Res. Int..

[38] Zhou Y.J. (2019). Individualized risk prediction of significant fibrosis in non-alcoholic fatty liver disease using a novel nomogram. United European Gastroenterol J.

[39] Tada T. (2021). Predictive value of cytokeratin-18 fragment levels for diagnosing steatohepatitis in patients with nonalcoholic fatty liver disease. Eur. J. Gastroenterol. Hepatol..

[40] Mikolasevic I. (2022). The accuracy of serum biomarkers in the diagnosis of steatosis, fibrosis, and inflammation in patients with nonalcoholic fatty liver disease in comparison to a liver biopsy. Medicina (Kaunas).

[41] Yip T.C. (2022). Asian perspective on NAFLD-associated HCC. J. Hepatol..

[42] Contreras D. (2023). Diagnostic accuracy of blood biomarkers and non-invasive scores for the diagnosis of NAFLD and NASH: systematic review and meta-analysis. Ann. Hepatol..

[43] Vilar-Gomez E., Chalasani N. (2018). Non-invasive assessment of non-alcoholic fatty liver disease: clinical prediction rules and blood-based biomarkers. J. Hepatol..

[44] Harrison S.A. (2020). A blood-based biomarker panel (NIS4) for non-invasive diagnosis of non-alcoholic steatohepatitis and liver fibrosis: a prospective derivation and global validation study. Lancet Gastroenterol Hepatol.

[45] Hagstrom H. (2017). Fibrosis stage but not NASH predicts mortality and time to development of severe liver disease in biopsy-proven NAFLD. J. Hepatol..

[46] Canivet C.M. (2022). Validation of the new 2021 EASL algorithm for the noninvasive diagnosis of advanced fibrosis in NAFLD. Hepatology.

[47] Younossi Z.M. (2021). Performance of the enhanced liver fibrosis test to estimate advanced fibrosis among patients with nonalcoholic fatty liver disease. JAMA Netw. Open.

[48] Seko Y. (2022). Diagnostic accuracy of enhanced liver fibrosis test for nonalcoholic steatohepatitis-related fibrosis: multicenter study. Hepatol. Res..

[49] Feng G. (2021). Machine learning algorithm outperforms fibrosis markers in predicting significant fibrosis in biopsy-confirmed NAFLD. J Hepatobiliary Pancreat Sci.

[50] Chauhan A. (2016). Diagnostic accuracy of hepatorenal index in the detection and grading of hepatic steatosis. J. Clin. Ultrasound.

[51] Cengiz M. (2014). Sonographic assessment of fatty liver: intraobserver and interobserver variability. Int. J. Clin. Exp. Med..

[52] Borges V.F. (2013). Sonographic hepatorenal ratio: a noninvasive method to diagnose nonalcoholic steatosis. J. Clin. Ultrasound.

[53] Paige J.S. (2017). A pilot comparative study of quantitative ultrasound, conventional ultrasound, and MRI for predicting histology-determined steatosis grade in adult nonalcoholic fatty liver disease. AJR Am. J. Roentgenol..

[54] Sasso M. (2016). Liver steatosis assessed by controlled attenuation parameter (CAP) measured with the XL probe of the FibroScan: a pilot study assessing diagnostic accuracy. Ultrasound Med. Biol..

[55] Chan W.K. (2014). Controlled attenuation parameter for the detection and quantification of hepatic steatosis in nonalcoholic fatty liver disease. J. Gastroenterol. Hepatol..

[56] Xia B. (2018). Feasibility and Efficacy of Transient Elastography using the XL probe to diagnose liver fibrosis and cirrhosis: a meta-analysis. Medicine (Baltim.).

[57] Taru M.G. (2023). How to identify advanced fibrosis in adult patients with non-alcoholic fatty liver disease (NAFLD) and non-alcoholic steatohepatitis (NASH) using ultrasound elastography-A review of the literature and proposed multistep approach. Diagnostics.

[58] Shen F. (2015). Impact of skin capsular distance on the performance of controlled attenuation parameter in patients with chronic liver disease. Liver Int..

[59] Siddiqui M.S. (2019). Vibration-controlled transient elastography to assess fibrosis and steatosis in patients with nonalcoholic fatty liver disease. Clin. Gastroenterol. Hepatol..

[60] Shima T. (2020). Diagnostic accuracy of combined biomarker measurements and vibration-controlled transient elastography (VCTE) for predicting fibrosis stage of non-alcoholic fatty liver disease. J. Gastroenterol..

[61] Newsome P.N. (2020). FibroScan-AST (FAST) score for the non-invasive identification of patients with non-alcoholic steatohepatitis with significant activity and fibrosis: a prospective derivation and global validation study. Lancet Gastroenterol Hepatol.

[62] Zhao G.D., Guo S.M., Xie Q. (2022). Diagnostic accuracy of fiborscan-AST score in nonalcoholic steatohepatitis with significant activity and fibrosis. J Clin Hepatol.

[63] Dyvorne H.A. (2016). Prospective comparison of magnetic resonance imaging to transient elastography and serum markers for liver fibrosis detection. Liver Int..

[64] Park C.C. (2017). Magnetic resonance elastography vs transient elastography in detection of fibrosis and noninvasive measurement of steatosis in patients with biopsy-proven nonalcoholic fatty liver disease. Gastroenterology.

[65] Forsgren M.F. (2020). Biomarkers of liver fibrosis: prospective comparison of multimodal magnetic resonance, serum algorithms and transient elastography. Scand. J. Gastroenterol..

[66] Loomba R. (2016). Novel 3D magnetic resonance elastography for the noninvasive diagnosis of advanced fibrosis in NAFLD: a prospective study. Am. J. Gastroenterol..

[67] Ajmera V., Loomba R. (2021). Imaging biomarkers of NAFLD, NASH, and fibrosis. Mol Metab.

[68] Tamaki N. (2022). Magnetic resonance elastography plus Fibrosis-4 versus FibroScan-aspartate aminotransferase in detection of candidates for pharmacological treatment of NASH-related fibrosis. Hepatology.

[69] Gallego-Duran R. (2016). Imaging biomarkers for steatohepatitis and fibrosis detection in non-alcoholic fatty liver disease. Sci. Rep..

[70] Dulai P.S. (2016). MRI and MRE for non-invasive quantitative assessment of hepatic steatosis and fibrosis in NAFLD and NASH: clinical trials to clinical practice. J. Hepatol..

[71] Dennis A. (2020). Correlations between MRI biomarkers PDFF and cT1 with histopathological features of non-alcoholic steatohepatitis. Front. Endocrinol..

[72] Ajmera V. (2018). Magnetic resonance imaging proton density fat fraction associates with progression of fibrosis in patients with nonalcoholic fatty liver disease. Gastroenterology.

[73] Noureddin M. (2022). MRI-based (MAST) score accurately identifies patients with NASH and significant fibrosis. J. Hepatol..

[74] Zhang R.N. (2018). Genome-wide analysis of DNA methylation in human peripheral leukocytes identifies potential biomarkers of nonalcoholic fatty liver disease. Int. J. Mol. Med..

[75] Gorden D.L. (2015). Biomarkers of NAFLD progression: a lipidomics approach to an epidemic. J. Lipid Res..

[76] Loomba R. (2015). Polyunsaturated fatty acid metabolites as novel lipidomic biomarkers for noninvasive diagnosis of nonalcoholic steatohepatitis. J. Lipid Res..

[77] Mayo R. (2018). Metabolomic-based noninvasive serum test to diagnose nonalcoholic steatohepatitis: results from discovery and validation cohorts. Hepatol Commun.

[78] Caussy C. (2019). Serum metabolites detect the presence of advanced fibrosis in derivation and validation cohorts of patients with non-alcoholic fatty liver disease. Gut.

[79] Jung Y. (2020). Circulating lipidomic alterations in obese and non-obese subjects with non-alcoholic fatty liver disease. Aliment. Pharmacol. Ther..

[80] Mowry C.J. (2021). Utility of metabolomic biomarkers to identify nonalcoholic fatty liver disease in liver transplant recipients. Transplant Direct.

[81] Masarone M. (2021). Untargeted metabolomics as a diagnostic tool in NAFLD: discrimination of steatosis, steatohepatitis and cirrhosis. Metabolomics.

[82] Marques V. (2021). Adiponectin, leptin, and IGF-1 are useful diagnostic and stratification biomarkers of NAFLD. Front. Med..

[83] Di Mauro S. (2019). Serum coding and non-coding RNAs as biomarkers of NAFLD and fibrosis severity. Liver Int..

[84] Becker P.P. (2015). Performance of serum microRNAs -122, -192 and -21 as biomarkers in patients with non-alcoholic steatohepatitis. PLoS One.

[85] Cai C. (2019). Circulating miRNAs as novel diagnostic biomarkers in nonalcoholic fatty liver disease: a systematic review and meta-analysis. Can J Gastroenterol Hepatol.

[86] Hendy O.M. (2019). The circulating micro-RNAs (-122, -34a and -99a) as predictive biomarkers for non-alcoholic fatty liver diseases. Diabetes Metab Syndr Obes.

[87] Luo Y. (2021). SOMAscan proteomics identifies serum biomarkers associated with liver fibrosis in patients with NASH. Hepatol Commun.

[88] Hou W. (2020). Proteomic screening of plasma identifies potential noninvasive biomarkers associated with significant/advanced fibrosis in patients with nonalcoholic fatty liver disease. Biosci. Rep..

